# In sickness and in health: group-living augments behavioural responses to food and predation risk for sick house finches (*Haemorhous mexicanus*)

**DOI:** 10.1098/rsos.251715

**Published:** 2025-11-12

**Authors:** Marissa M. Langager, Alicia G. Arneson, Dana M. Hawley

**Affiliations:** ^1^ Department of Biological Sciences, Virginia Tech, Blacksburg, VA, USA

**Keywords:** behavioural tolerance, group living, directly transmitted pathogen, foraging behaviour, anti-predator behaviour, house finch, *Mycoplasma gallisepticum*

## Abstract

Group-living provides many fitness benefits for individual members, including improved foraging and predator vigilance. If such benefits are especially pronounced for sick members, group-living can act as a form of behavioural tolerance by offsetting mortality costs of infection. We experimentally tested this possibility by examining whether group-living impacts foraging and anti-predator behaviours in house finches (*Haemorhous mexicanus*) with or without conjunctivitis caused by *Mycoplasma gallisepticum*. We varied both group-living (single-housed or group-housed) and infection (*M. gallisepticum*-inoculated or sham-inoculated) and performed four behavioural assays at peak infection: two assessing how birds respond to foraging opportunities and two assessing responses to predation threats. Both social treatment and disease status influenced most measured behaviours, with single-housed, diseased birds consistently the least responsive to foraging opportunities and predation threats. While group-living also benefited healthy individuals (e.g. led to faster responsiveness) in most behavioural assays, our results suggest that diseased birds particularly benefit from group-living. Further, detected behavioural differences with group-living were not explained by effects of sociality on disease severity or pathogen load, which did not differ with group-living. By augmenting behaviours key to survival during infection, group-living may act as a form of behavioural tolerance for social species, with important implications for transmission dynamics.

## 1. Introduction

For gregarious species, maintaining social groups is vitally important for diverse aspects of individual fitness. Despite potential costs of group-living such as competition [1,2] and increased pathogen risk [3], sociality has evolved multiple times across diverse taxa [4], suggesting that benefits of sociality outweigh its costs for many animals. The key benefits of group-living, which include increased food acquisition and decreased time spent on predator vigilance by individual group members [5–7], may be especially important for individuals actively experiencing acute infections [8], who might rely heavily on healthy group members for fitness benefits such as food acquisition and predator detection [9]. Nonetheless, few studies have explicitly examined the potential benefits of group-living for actively infected individuals (but see [10]). Determining whether group-living particularly benefits infected individuals is key for understanding social preferences during infection as well as its possible impacts on population-level transmission dynamics.

Pathogen infections in vertebrates typically result in sickness behaviours characterized by overall reductions in activity level and social interactions, which may limit the ability of infected animals to behave optimally within their environment [11,12]. Animals displaying sickness behaviours are often more susceptible to predation [13] and can have difficulty locating or partaking in food resources [10]. Results from several studies suggest that group-living can reduce the fitness costs of infection for hosts through several different possible mechanisms, including increased predator vigilance by conspecifics, territory defence and improved foraging success [9,10]. Thus, for gregarious species, infected individuals may utilize group-living as a form of behavioural tolerance, defined here as using behaviour to offset the fitness costs incurred during infection [8].

Both foraging and anti-predator benefits of group-living require the transmission of information among group members [2]. Social groups may provide infected individuals with information they could not acquire otherwise, such as locations of foraging sites or predator presence. One mechanism that utilizes this information transfer to benefit group-living individuals is the ‘many-eyes effect’, where groups provide more total individuals to scan for predators, allowing group members both to allocate more of their daily time budget towards feeding and to respond more quickly to predators [14]. For example, flock size influences the latency to detect and respond to a predator in several bird species, with birds in larger versus smaller flocks responding more quickly to a perceived predator threat [15]. The importance of group-living has also been demonstrated for infected animals, with herding gazelle harbouring gastrointestinal nematodes showing increased foraging efficiency due to the dilution of individual anti-predator vigilance [9]. Information transfer and the presence of ‘many eyes’ that are associated with group-living could play a key role in offsetting the fitness costs incurred by sickness behaviours such as lethargy [16], which are hypothesized to help acutely infected animals divert energy towards immunity [12], but can also lower an individual’s ability to maintain other aspects of fitness [17].

Host–pathogen systems where acute infections occur during periods of group-living provide key opportunities for directly testing whether grouping behaviour can offset fitness costs of infection, thereby acting as a form of behavioural tolerance. One such example is the naturally occurring host–pathogen system of house finches (*Haemorhous mexicanus*) and their bacterial pathogen *Mycoplasma gallisepticum* (MG), which leads to severe conjunctivitis in infected birds [18]. House finches are a gregarious North American songbird species that form large foraging flocks, particularly during the autumn and winter, congregating around bird feeders which facilitate MG spread [19] and seasonal outbreaks of disease [20].

Group-living can potentially offset the costs of MG infection in several ways. First, flocking could offset the higher predation risk experienced by infected individuals, which likely constitutes the primary source of MG-mediated mortality in wild birds [21]. MG-infected house finches show lethargy [18], the potential for obscured vision when conjunctivitis is severe and reduced anti-predator responses relative to healthy birds [13], all of which may increase their likelihood of depredation. Group-living may help offset the cost of decreased anti-predator vigilance during infection by alerting infected individuals to predators and, consequently, increasing foraging time, through the ‘many-eyes effect’ [15,22], allowing birds in a flock a higher likelihood of surviving infection. Finally, group-living itself may result in suppression of sickness behaviours for sick individuals surrounded by uninfected conspecifics, leading sick individuals to be more active when in a group than if isolated, an effect seen in zebra finches and hypothesized to decrease behavioural costs associated with infection [23]. Because infection with MG is associated with an overall reduction in activity levels [18], suppression of sickness behaviours may allow infected group members to take better advantage of the benefits associated with group-living, increasing their chances of surviving infection.

Infection with MG also impacts the social preferences of house finches in ways consistent with group-living benefiting infected finches. When given the choice to associate with a flock or alone, house finches experimentally inoculated with MG preferred to eat in association with a flock significantly more strongly than did uninfected birds [24]. This strengthening of social preference during infection may indicate that benefits of group membership are especially important for infected birds in this system. Here, we assessed the two primary benefits of group-living in songbirds (foraging and anti-predator behaviours) to test whether infected house finches benefit behaviourally from group-living. To elucidate the role of group-living on foraging behaviours and predator responses of MG-infected house finches, we conducted a 2 × 2 factorial design in which wild-caught, but captive-held, house finches were assigned to a group-living treatment (group-housed or isolated) and an infection treatment (inoculated with MG or sham-inoculated with sterile media) and then tested via four behavioural assays to measure responses to both foraging and predation challenges. We also examined whether social treatment altered the severity of infection (pathogen load) or disease (conjunctivitis severity) that experimentally infected birds experienced, because such effects could contribute to detected behavioural differences in response to group-living [13]. In line with the ‘many eyes’ hypothesis, we predicted that infected birds housed in groups would respond more rapidly to foraging opportunities and predation threats than single-housed infected birds, despite birds in both social treatments harbouring equivalent pathogen loads and conjunctival swelling.

## 2. Methods

### 2.1. Experimental design

Our experiment varied group-living treatment (single-housed or group-housed in a flock of five total birds) and infection status (sham- or MG-inoculated) for 32 ‘focal’ birds in a fully factorial design (*n* = 8 per social housing/infection status combination). Each group-housed focal bird was co-housed with four flockmates, one of which (per flock) was used as a sham control to better account for potential variability among flocks. To control for potential effects of cage size on behavioural responses in the four assays, all birds, regardless of social group treatment, were housed in identically sized large flight cages. As noted below, because MG transmission from focal infected birds to healthy flockmates occurred rapidly in group-housed treatments, in some cases prior to our behavioural assays, we also collected behavioural data from birds that became MG-infected via transmission for certain assays (see §2.4), resulting in the use of data from all birds (focal birds plus additional flockmates from group-housed treatments) for some assays.

We assayed behavioural responses (foraging and anti-predator behaviours) using four distinct assays conducted on different days during the window of peak infection (days 10–20 [25]). To complete the required behavioural tests during this window, we split the experiment into two temporal batches (one month apart, with each individual bird only included in one unique batch). Experimental batch 1 (*n* = 28 total birds; *n* = 16 focal birds) ran from late June 2022 to mid-July 2022. Experimental batch 2 (*n* = 28 total birds; *n* = 16 focal birds) ran from late July 2022 to mid-August 2022. Both batches contained equal proportions of all treatment combinations.

### 2.2. Study subjects and capture

Fifty-six hatch-year house finches were captured from Radford or Blacksburg, Virginia, USA in May–July 2022 using baited wire-mesh traps and housed initially in pairs (see electronic supplementary material). Because the study examined responses to MG infection, we ensured all birds used in the study did not have prior exposure to MG by examining birds for clinical signs of MG at least three times over a quarantine period of 14 days post-capture. We also ensured the 56 birds used were seronegative for MG prior to experimental infection, which was determined by testing blood plasma with an anti-MG IgY ELISA kit [26]. Based on prior work demonstrating that hatch-year house finches show no sex differences in social affiliative behaviour [24] and because sex differences in plumage were not yet present in birds at the time of the study, we did not sex birds for this experiment.

### 2.3. Social group treatment and housing

Ten days before inoculation, all birds were assigned to a social group treatment, either group-housed (a flock of five birds housed together) or single-housed. Each group-housed cage contained one MG-inoculated and one sham-inoculated focal individual (see §2.4). All five flock members were housed together in a large flight cage (100 × 34 × 34 cm^3^) and provided food ad libitum across three food dishes to account for the increased number of birds present within the cage. Single-housed birds were provided food ad libitum in a single food dish. House finches were given a unique set of colour bands according to their social group treatment before being moved into their assigned group housing. Each flight cage was given a unique cage ID number and equipped with three perches and a cardboard box in the upper right corner to provide a refuge area. While this refuge area was specifically installed for use in the predator assay, cages were outfitted with the boxes prior to housing birds, to minimize disturbance as well as acclimatize birds to the presence of the refuge area.

A total of seven housing rooms were used for each temporal batch, and all housing rooms were temperature-controlled and kept on a 12L : 12D photoperiod throughout the experiment. Because even perceptions of social isolation can influence immunity and other physiological responses in many vertebrates [27], all single-housed, infected birds were housed alone in a room to simulate true social isolation. This allowed us to robustly test whether social isolation influences host responses to disease (pathogen loads and pathology) that could influence the behavioural responses of interest here. However, this need for true social isolation created space constraints that influenced the housing of the other treatment groups. For the single-housed sham-inoculated individuals, for which disease responses were not relevant because all birds remained healthy, birds were housed singly in cages in a room containing four cages, which were separated by barriers so that each cage was visually, but not auditorily, isolated from each other. Further, two cages of flock-housed individuals were kept within the same room during each batch of the experiment and were separated by a barrier so that each flock was visually, but not auditorily, isolated from one another throughout the experiment. Overall, this set-up allowed us to robustly test the key questions of interest within the confines of available housing rooms, while minimizing temporal confounds that would have resulted had we not run all treatment groups concomitantly.

### 2.4. Dominance trials and infection treatment

Because an individual’s social status within a group can impact how behaviours are expressed during infection [28], we assessed the social status of each flock member prior to experimental infection to determine which bird in each flock would be inoculated with MG. To assess social status without human interference, we video-recorded each flock for 60 min and observed dyadic interactions around a single food dish (see electronic supplementary material for details). We assigned each bird a social rank from 1 to 5, with the bird having won the most dyadic interactions at rank 1. To avoid inoculating the most dominant or submissive bird in a flock, we assigned birds at rank 3 (the most central social status) as the focal birds for the MG-inoculated, group-housed treatment (*n* = 8). Sham-inoculated treatments within the flock were randomly assigned to either the second- or fourth-ranked flock member using a coin toss (*n* = 8). The remaining three birds in a flock were not assigned to a specific infection treatment, serving as ‘flockmates’.

Though only two birds in each flock served as focal individuals (one MG-inoculated and one sham-inoculated per flock) and were directly inoculated on experiment day 0, there was rapid MG transmission among group-housed birds such that by experiment day 11, 4/8 group-housed sham-inoculated birds and 12/24 of the birds designated as flockmates were displaying clinical signs of MG infection; on experiment day 15, the number of flockmates displaying clinical signs of MG had increased to 14/24 flockmates. Because the disease status of an individual’s group members can impact their behaviour [29], we recorded the disease status (diseased or healthy) for each bird at the time of each behavioural assay and included all birds, with their current disease status, in some analyses. For the purposes of our analyses, ‘healthy’ birds are those without clinical signs of MG and ‘diseased’ birds are those displaying signs of MG infection. We use disease status rather than infection status (determined via qPCR) because (i) disease status is a highly reliable indicator of infection for birds of unknown MG status (capturing 97% of positive cases in past work [30]) and (ii) we had higher temporal sampling resolution for disease status versus infection status in our study (see §2.5), allowing us to more robustly determine disease status versus infection status at the time of the assay for a given individual.

### 2.5. Inoculation and disease monitoring

To ensure that birds developed sufficiently high disease severity to influence foraging and anti-predator behaviours, we used an MG strain known to be virulent in this finch population [24,31], collected from North Carolina, USA, in 2006 (NC2006, 2006.080-5 4P 7/26/12, David H. Ley, NC State University, College of Veterinary Medicine, Raleigh, NC, USA). On experimental day 0, birds assigned to a given treatment were inoculated bilaterally in the conjunctiva via droplet instillation of 70 µl of MG in Frey’s media (MG-inoculated treatment: single-housed *n* = 8, group-housed *n* = 8) or with the same volume of sterile media alone (sham-inoculated treatment: single-housed: *n* = 8, group-housed *n* = 8). To ensure that handling stress was uniform across all birds in the experiment, the remaining 24 flock-housed birds that were not assigned to either the infection or sham-inoculated treatment were handled in the same manner on experiment day 0 as the birds in an assigned focal treatment, but the clean pipette was only held above the eye briefly.

Disease severity was monitored weekly for all birds until experiment day 16 by scoring conjunctivitis on a 0–3 scale per eye, with scores of 3 displaying the most severe conjunctivitis [26]. If weekly conjunctival scoring did not occur within ±2 days of a behavioural assay, we reassessed eye score after the behavioural assay was completed for the day. Within each sampling day, disease severity scores were summed across eyes to calculate a combined severity score of 0–6. In addition to scoring disease severity, we swabbed the conjunctiva twice, on experiment days 7/8 and 15/16 (see §2.6.2 for why sampling occurred over two days), to quantify MG load. Swabs were placed into 300 µl tryptose phosphate broth (TPB) and stored at −20°C until extraction using a Qiagen 96 DNeasy Blood and Tissue Kit; a probe-based qPCR was used to determine the amount of MG in each sample [26].

### 2.6. Behavioural assays

#### 2.6.1. Feeder assays

Birds were video recorded in their home cages on two consecutive days for two different feeder behavioural assays. First, on experiment day 11, we assessed latency to approach and use the normal feeder dish in its usual location within the home cage (the front left corner) following removal of the dish to simulate food disturbance. Second, on experiment day 12, we assessed latency to approach and used a novel, brightly coloured feeder that was placed in a location different from that of their normal food dish, hanging near the back right side of the cage. To standardize motivation to feed, we removed all normal food dishes from the cages 30 min before lights out the night before the assay and only returned food at the start of the assay, 30 min after the lights had turned on for the day (totaling 60 min of daytime food restriction). Video was recorded for 45 min starting at 07.00, when food was returned to the cage. Both the normal and novel food dishes were open cups, such that birds could visually identify their usual food source within the dish. Each dish also had a small perching area for use while eating.

All videos were scored by a single observer using BORIS [32], with perching and eating behaviours coded continuously (see electronic supplementary material, table S1, for ethogram) throughout the video. While it was impossible for the observer to be blind to social group treatment, observers were blind to infection treatment during video scoring.

#### 2.6.2. Capture evasion assay

To determine how long a bird could evade capture by a potential survival threat, we quantified the time it took to capture each bird from its cage, with all captures throughout the study done by a single person unfamiliar with the goals of the study. On experiment days 7/8 and 15/16, all focal birds in both the MG-inoculated (single-housed: *n* = 8; flock-housed: *n* = 8) and sham-inoculated treatments (single-housed: *n* = 8; flock-housed: *n* = 8) were captured as rapidly as possible. The total time that each bird was able to evade capture by the observer was recorded as the time elapsed between opening the cage door to the time the bird was securely caught. Because all group-housed treatment cages contained a single bird in the assigned MG-inoculated treatment and a single bird in the assigned sham-inoculated treatment, we spread our capture trials across two consecutive days, with one focal bird in each flock captured first per day, ensuring that we accurately assessed the fastest capture time for each of the flock’s focal birds. Birds from both the single- and group-housed social group treatments were randomly assigned to be sampled on one of the two consecutive days, so that each focal bird was only timed for capture evasion and sampled once in a weekly period. Time to evade capture was not recorded for any of the birds within the group-housed treatment that were assigned as ‘flockmates’.

#### 2.6.3. Simulated aerial predator assay

We used a simulated aerial predator (a hawk silhouette kite mounted on a zipline) to assess response time to a mock predator flyover. To standardize the predator mounting and zipline, all aerial predator assays were conducted in a single assay room within the same facility. Each home cage was transferred from its captive room to the predator assay room, approximately 3–6 m from each housing room. Tests were carried out only once for each treatment cage during the study, and for only one cage at a time, such that a given cage was the only one present in the assay room when the mock aerial predator was released. To ensure all cages were tested during peak foraging times (07.00–10.30), tests were carried out over three days (experiment day 13, 14 or 15). All birds were food restricted for 60 min prior to their assay start.

To prevent prolonged visual access to the simulated predator during the trial, a temporary black plastic sheet was clipped to the back of the cage prior to it being moved into the predator assay room. Further, to prevent birds from seeing the simulated predator before the assay began, all cages were moved into the predator assay room before lights were turned on. After placing each cage in the room, video recording began and then the lights were turned on from outside the room. Overall, each cage was recorded for 35 min, with four cages tested each morning from 07.00 to 10.00.

Approximately 30 s after the tester left the room, a 5-s playback from a common predator of finches, a Cooper’s hawk (*Accipiter cooperii*), was played every 15 s over the course of 1 min. During the last 5-s playback section, a kite representing a bottom-up view of a hawk was released, causing it to fly down a zipline system over the birds being tested. Each bird’s response during the simulated predator flyover (from the time the hawk was released to the time it landed against the far wall, out of sight) was recorded (see electronic supplementary material, table S2, for ethogram). In addition to their response during the simulated hawk flyover, we also recorded the total time each individual spent immobile (for a minimum 5-s duration) during the 30-min ‘recovery’ period after the predator flyover. All videos were scored by a single observer who was blind to disease status, but not social treatment, using BORIS [32].

### 2.7. Statistical analysis

All analyses were completed in R v. 4.3.2 [33] and plots were made in ggplot2 [34]. Because transmission within the group-housed treatment cages resulted in some sham-inoculated focal birds showing clinical signs of conjunctivitis by the time of a given assay, we used the actual disease status (diseased or healthy) of a bird at the time of each behavioural assay in all analyses. For the time of capture assay, only focal birds (*n* = 32) and their disease status at the time of assay were included in the analysis because capture times were not recorded for flockmates. For all other behavioural assays, because rapid transmission (see electronic supplementary material, table S3) limited the sample sizes of healthy control birds (reduced from the eight original sham controls), we included untreated flockmates (with their actual disease status at the time of the assay) in analyses of both feeder assays and the aerial predator assay, with the unique cage ID of each flock included as a random effect for each individual (see below). To ensure that differences in transmission speed and resulting disease prevalence among flocks did not influence our results, we performed a subset of additional analyses categorizing flocks as lower (2–3 diseased birds per flock) or higher (4–5 diseased birds per flock) prevalence at the time of the assay (see electronic supplementary material, figures S2–S4).

For all behavioural analyses, we included disease status (diseased or healthy) at time of assay, social treatment (group-housed or single-housed), and the interaction of social treatment and individual disease status as predictor variables. Interactions were removed from final models if *p* > 0.1. The significance of the predictor variables in our models was tested using the ANOVA function (for generalized linear mixed models (GLMMs); car package [35]) or Anova.clmm function (RVAideMemoire package [36]) to generate either type II likelihood-ratio tests if interactions were not significant or type III tests in models with significant pairwise interactions.

Cage ID was included as a random intercept in all models, with variance associated with cage ID reported in electronic supplementary material, table S5. We also calculated the intra-class correlation coefficient (ICC) as a metric of between-cage variance. The ICC was calculated as


$$ICC=\frac{\sigma_{\alpha }^{2}}{\sigma_{\alpha }^{2}+\sigma_{\varepsilon }^{2}},$$

where $\sigma_{\alpha }^{2}$ is the random effect variance that accounts for variance between cages or birds, depending on the model, and $\sigma_{\varepsilon }^{2}$ is the residual variance (the variance that exists due to unaccounted for sources). Given this form, for our study, a lower ICC indicates a higher degree of agreement between cages or birds due to a smaller variance coming from our random effect compared to that coming from other sources. The random effect variance was taken from the modelling output in R, and the residual variance was also taken from the modelling output for LMMs and GLMMs. The residual variance is constrained to be $\pi^{2}/3$ for cumulative link mixed models (CLMMs).

As an additional check of whether the non-independence of flockmates within a group influenced our results, we performed most of the below analyses on both the full dataset as well as a subset dataset that included only one diseased bird per flock (the focal birds) and one healthy bird per flock (because several sham control birds became diseased as a result of transmission, the healthy bird per flock in our subset data was randomly selected from among the birds that were still healthy in a given flock at the time of the assay; electronic supplementary material, table S4). Results from the subset data are presented in the electronic supplementary material.

#### 2.7.1. Foraging assay analyses

**Figure 1.**
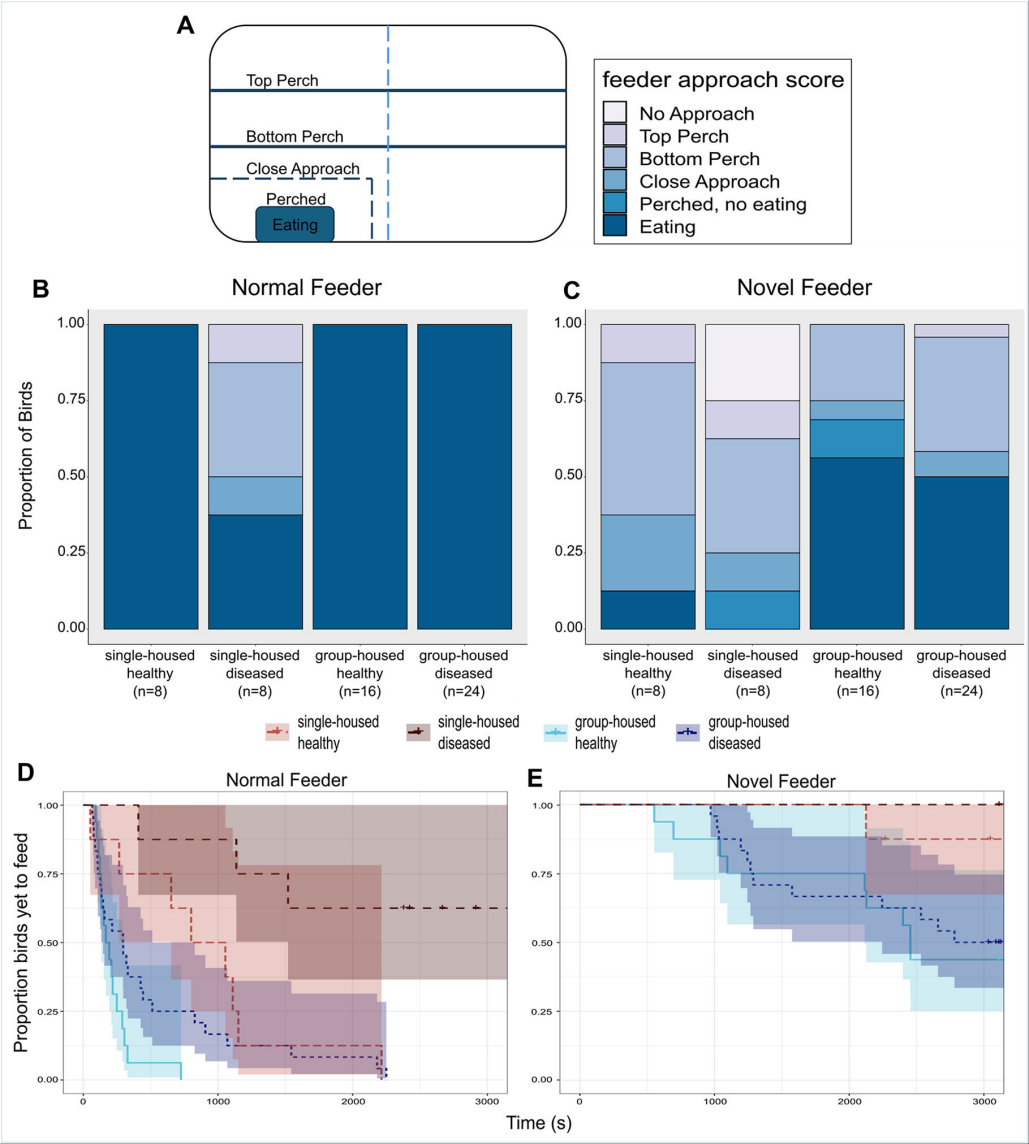
(A) A top-down view of the normal feeder assay. A feeder approach score from 0 to 5 designates how close each bird came to eating from the food dish. Approach scores were only assigned when the bird was in the half of the cage containing the food dish. (B) When offered their normal feeder type after 60 min of food restriction, only birds in the single-housed diseased category showed any variation in response, with 38% of birds eating from the normal feeder, versus 100% of all group-housed birds and single-housed healthy. (C) Following 60 min of food restriction, single-housed house finches were significantly less likely to eat from a novel feeder than group-housed birds, regardless of disease status. (D) Single-housed birds were slower to feed from their normal food dish than group-housed birds, and (E) single-housed birds had a higher latency to approach and eat than birds in flocks.

Using data from our feeder assays, we asked how an individual’s disease status and group-living treatment influenced the likelihood to approach and eat from their normal feeder following disturbance, and the likelihood to eat from a brightly coloured, novel feeder. We quantified willingness to approach each feeder type by assigning a score from 0 to 5 to denote how closely an individual bird came to eating from the feeder (figure 1A), with a score of 0 indicating birds that stayed >30 cm from the feeder (considered to be ‘no approach’) and a score of 5 assigned to birds who ate from the feeder during the assay. The approach scores recorded during the novel feeder assay were treated as ordinal factors and used as the response variable in CLMMs in the ordinal package [37]. For our normal feeder assays, because the distribution of response scores (with only single-housed birds showing any variation in score) did not allow a CLMM to converge, we used a Fisher’s exact test comparing approach scores across the four treatment combinations.

To complement the above analyses, we used mixed effect Cox models (coxme package [38]) to analyse latency to approach and eat from the feeder for each assay. For both the normal feeder and novel feeder assays, a binary measure denoting whether or not a bird had eaten from the feeder (0—did not eat; 1—did eat) as well as the latency to feed was used as response variables in our analysis. Finally, for both foraging assays, we performed Wilcoxon rank-sum tests to determine whether healthy or diseased birds in the flock-housed treatments were more likely to eat before other flockmates from either feeder (normal or novel feeder) during a given assay. This analysis was limited to the flock-housed birds only, with feeding order within each flock ranked from 1 to 5 based on latency values.

#### 2.7.2. Anti-predator behaviour analyses

Next, we examined how group-living and disease status impact anti-predator behaviours in the face of a perceived predation threat (either capture by a human or a simulated aerial predator flyover). Latency to evade capture was used as the response variable in a GLMM (using Gamma error distribution linked with the log function) for capture evasion on day 7/8. We did not analyse time to capture on experiment day 15/16 because, by that point in the study, sufficient transmission to flockmates had occurred that sample sizes were too unbalanced to examine the interaction between group-living and disease status (electronic supplementary material, table S3).

The latency to respond to the aerial predator flyover was determined by subtracting the time the hawk started to ‘fly’ from the time of the bird’s first recorded response during the flyover (for behaviours that we considered a predator response, see electronic supplementary material, table S2). The latency time recorded for birds who did not exhibit a response during the hawk flyover was the time that the hawk hit the back wall (and was no longer visible to birds in the cage), indicating that these birds did not react during the entirety of the hawk flyover. This time to first response was divided into quartiles, with the birds that were slowest to respond (or did not respond at all) to the predator flyover sorted into the lowest quartile (quartile 4) and the birds who responded most quickly into the highest (quartile 1). These quartiles were used as ordinal factors which were the response variable in a CLMM [37].

In addition to the CLMMs, we used mixed effect Cox models (coxme package [38]) to analyse the latency to respond to the predator flyover. Similar to the Cox models analysing latency to feed, a binary measure of whether or not a bird responded to the predator as well as the latency to their response was used as response variables in our models.

To analyse a potential proxy for sickness behaviours, as well as the degree to which individuals resumed activity after the aerial predator flyover, we measured time spent immobile in the 30 min directly after the aerial predator flyover as a proxy for sickness-induced lethargy. The proportion of time spent immobile was used as the response variable in an LMM, which was weighted by the total time of the assay.

#### 2.7.3. Disease and pathogen severity

Within the MG-inoculated treatment only (*n* = 16 focal birds), we tested whether group-living treatment influenced either disease severity or pathogen load using separate LMMs. Because both disease severity and pathogen load were taken over multiple sampling periods, each LMM incorporated bird ID as a random effect. For this analysis alone, cage ID was not included as a random effect because the 16 infected focal birds were all in independent cages.

## 3. Results

### 3.1. Feeder assays

The combination of social treatment and individual disease status influenced the likelihood that house finches approached and ate from their normal feeder following disturbance (Fisher’s exact test, *p* < 0.0001). Specifically, only 37% (3/8) of single-housed, diseased birds ate from their normal feeder during the assay, whereas 100% of birds in all other treatment groups ate during the assay (group-housed diseased: 24/24; group-housed healthy: 16/16; single-housed healthy: 8/8; figure 1B). In terms of latency to feed at the normal dish, social treatment and disease status both had independent effects in our model. Group-housed birds had a lower latency to eat from the feeder than single-housed birds, and healthy birds had a lower latency to start feeding than diseased birds (figure 1D; *n* = 56; social treatment likelihood ratio *χ*^2^ = 20.16, d.f. = 1, *p* < 0.0001; disease status likelihood ratio *χ*^2^ = 11.22, d.f. = 1, *p* < 0.001; mean time to eat: group-housed healthy, 220.72 s; group-housed diseased, 529.02 s; single-housed healthy, 912.77 s; single-housed diseased, 2083.95 s).

Only group-living treatment significantly predicted how likely a bird was to approach and eat from the novel feeder, with group-housed birds more likely to eat from the novel feeder than single-housed birds, independent of disease status (figure 1C; *n* = 56; social treatment likelihood ratio *χ*^2^ = 9.90, d.f. = 1, *p* < 0.002; disease status likelihood ratio *χ*^2^ = 2.07, d.f. = 1, *p* = 0.15). Notably, however, none of the birds in the single-housed diseased group ate from the novel feeder, though one bird did perch on it but never ate (group-housed diseased: 12/24; group-housed healthy: 9/16; single-housed diseased: 0/8; single-housed healthy: 1/8). The patterns for latency to feed from the novel feeder largely mirrored results from the normal feeder assay, with group-housed birds generally (though not significantly at *α* = 0.05) displaying a lower latency to locate and eat from the feeder than single-housed birds (figure 1E; *n* = 56; social group treatment likelihood ratio *χ*^2^ = 3.14, d.f. = 1, *p* = 0.076), and diseased birds having a significantly longer latency to eat from the novel feeder than healthy birds (disease status likelihood ratio *χ*^2^ = 4.31, d.f. = 1, *p* = 0.038). Interestingly, the significant effect of disease status appears driven by the universal lack of eating from the novel feeder in single-housed, diseased birds (mean time to eat: group-housed diseased, 2388.98 s; group-housed healthy, 2312.026 s; single-housed diseased, N/A; single-housed healthy, 2922.008 s). However, the main effect of disease on latency to eat from a novel feeder was the only foraging result that was no longer significant when we analysed the subset data (see electronic supplementary material), suggesting a lower weight of evidence for this result.

During the novel feeder assay, healthy birds were significantly more likely to feed earlier in rank than diseased birds (*W* = 277, *p* = 0.012, *n* = 24 diseased birds, *n* = 16 healthy birds). However, infection treatment did not influence feeding order in the normal feeder assay (*W* = 216, *p* = 0.51, *n* = 24 diseased birds, *n* = 16 healthy birds).

### 3.2. Capture evasion

**Figure 2.**
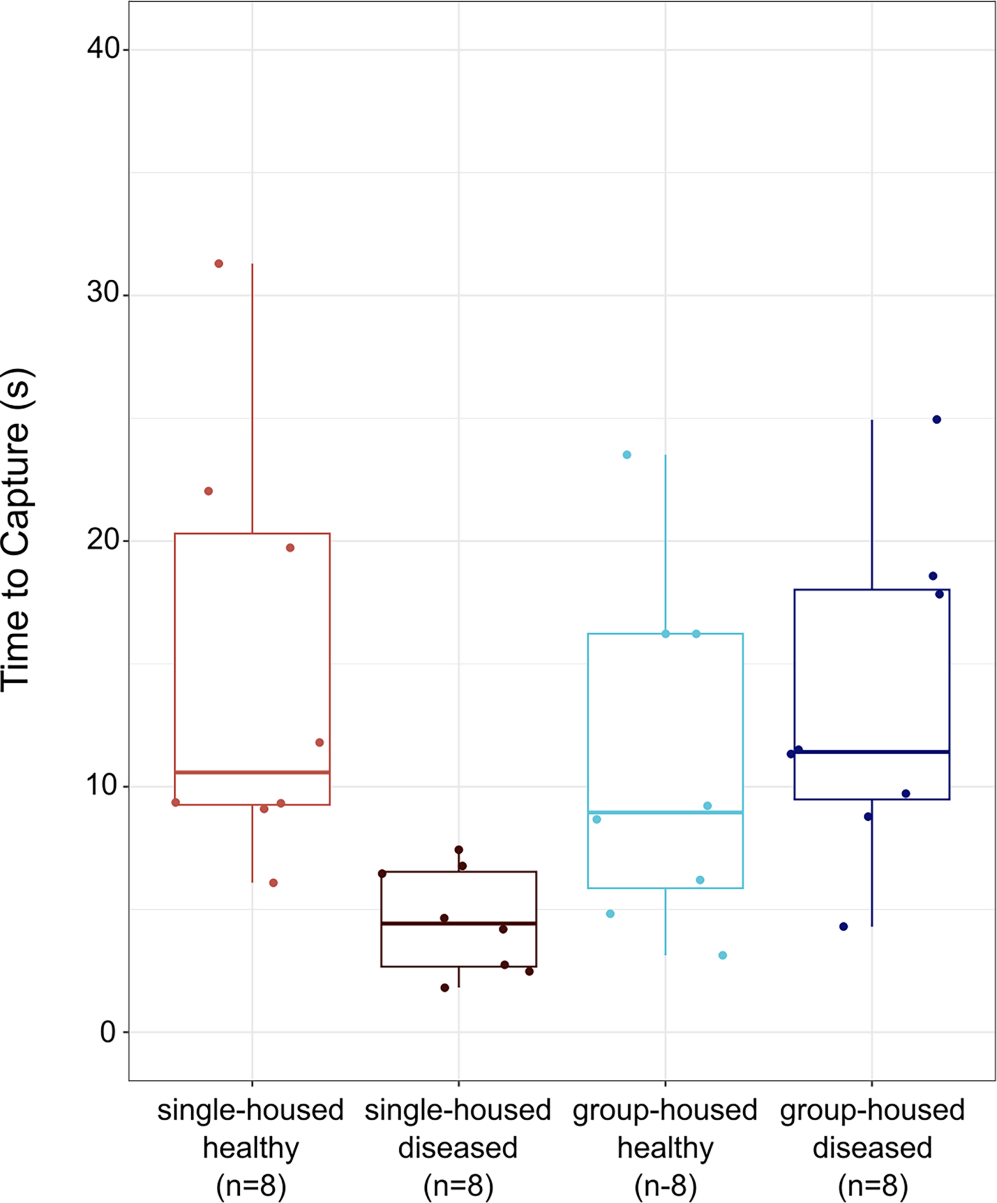
Single-housed house finches with mycoplasmal conjunctivitis were hand-caught significantly faster than group-housed or single-housed healthy finches.

The interaction between an individual’s social group treatment and their disease status influenced the speed at which they were captured on day 7/8, such that single-housed diseased birds were captured significantly more quickly (1.82–7.44 s) by a human than group-housed healthy birds (3.13–23.52 s) (figure 2; *n* = 32; social treatment × disease status *χ*^2^ = 14.27, d.f. = 1, *p* < 0.001). As a main effect, social treatment did not predict time to capture (*χ*^2^ = 0.76, d.f. = 1, *p* = 0.38). However, disease status as a main effect did significantly predict capture time (*χ*^2^ = 15.1, d.f. = 1, *p* < 0.001).

### 3.3. Aerial predator assay

**Figure 3.**
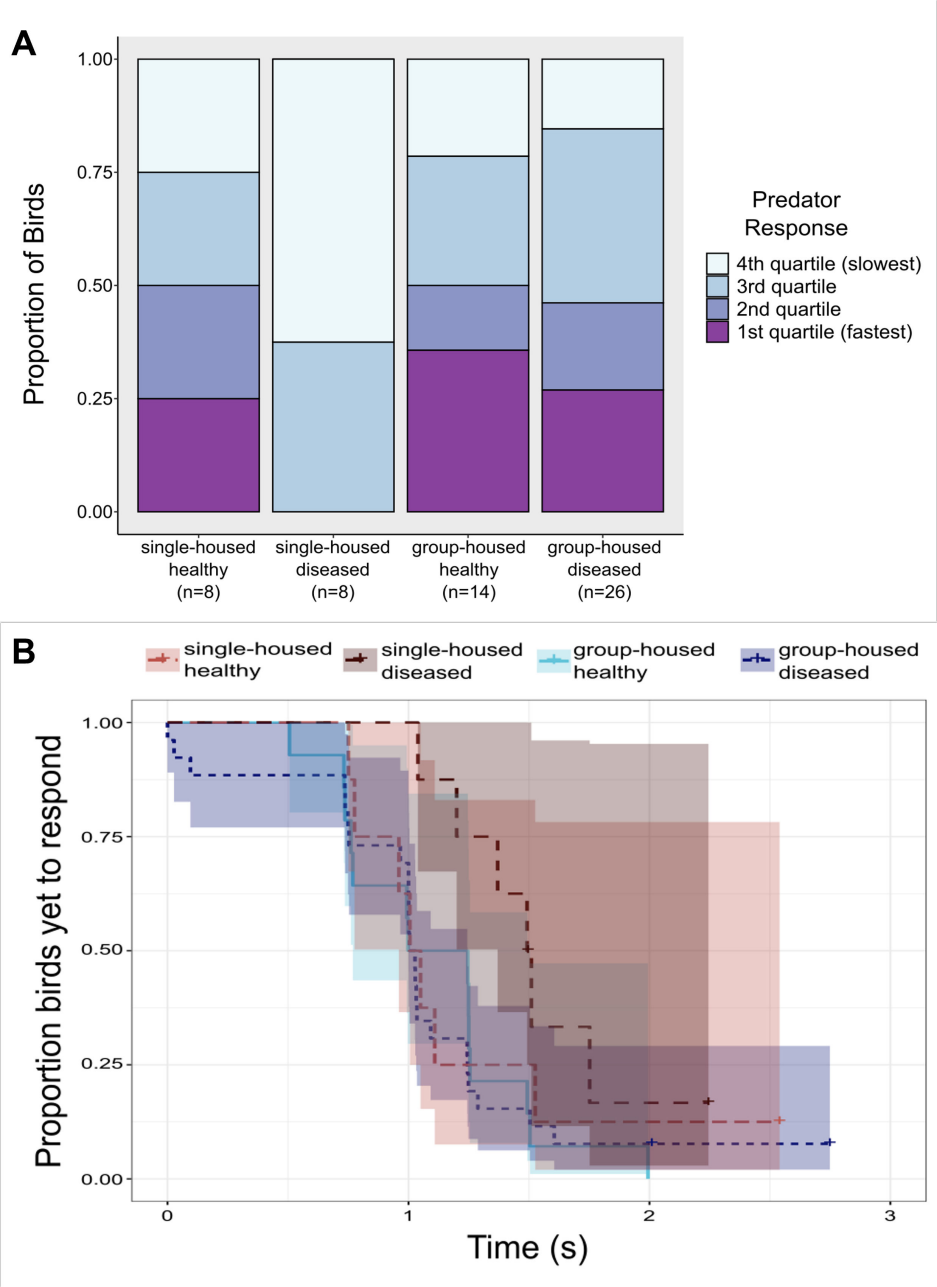
(A) The interaction between social group treatment and disease status significantly predicted how quickly a house finch responded to a simulated predator flyover, with group-housed birds more likely to respond quickly to the mock predator compared to single-housed, diseased birds. Here, reaction time is analysed as quartile scores (see §2). (B) Single-housed, diseased birds were slower to respond to the predator than group-housed birds or single-housed healthy birds.

Both social treatment and a bird’s disease status interacted to predict how quickly an individual bird responded to the mock predator flyover, as measured by quartile scores (figure 3A; *n* = 56; CLMM social treatment × disease status likelihood ratio *χ*^2^ = 3.94, d.f. = 1, *p* = 0.047), with single-housed diseased birds responding most slowly (mean = 1.51 s) relative to all other treatments (figure 3B). Neither social treatment as a main effect (likelihood ratio *χ*^2^ = 0.00, d.f. = 1, *p* = 1.00) nor disease status (likelihood ratio *χ*^2^ = 0.00, d.f. = 1, *p* = 0.99) predicted quartile scores of latency to respond to the predator flyover. In our Cox models analysing raw individual latency times, we found that, while not quite significant, our single-housed birds were moderately slower to respond to the mock predator (likelihood ratio *χ*^2^ = 3.58, d.f. = 1, *p* = 0.059), though individual disease status had no effect on latency to respond (likelihood ratio *χ*^2^ = 0.39, d.f. = 1, *p* = 0.53).

**Figure 4.**
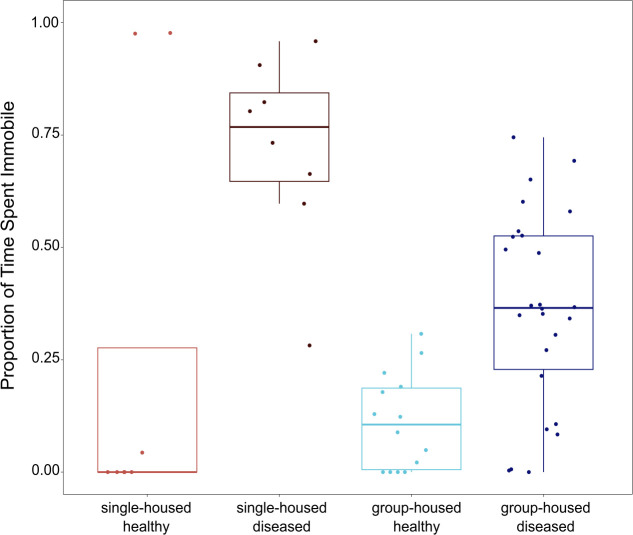
Single-housed diseased birds spent more time immobile during the 30 minutes following the mock predator release than all group-housed birds and single-housed healthy birds.

The interaction of social group treatment and disease status significantly predicted the time a bird spent immobile during the 30-min recovery period after aerial predator flyover (Wald *χ*^2^ = 3.69, d.f. = 1, *p* = 0.05), with single-housed diseased birds spending more time immobile than birds in any other treatment category (figure 4). Social group treatment as a main effect did not predict the time spent immobile (Wald *χ*^2^ = 0.35, d.f. = 1, *p* = 0.55). However, we did find a significant main effect of disease status on time spent immobile (Wald *χ*^2^ = 10.70, d.f. = 1, *p* = 0.001).

### 3.4. Disease severity and pathogen load

Within MG-inoculated focal birds, there was no significant effect of social treatment on either disease severity (electronic supplementary material, figure S1; *n* = 16; social treatment *χ*^2^ = 0.173, d.f. = 1, *p* = 0.68) or pathogen load (electronic supplementary material, figure S1; *n* = 16; social treatment *χ*^2^ = 0.067, d.f. = 1, *p* = 0.80).

### 3.5. Variation among cages

The ICC was used to assess the consistency across cages and/or birds within our experimental set-up. For models with repeated observations within each cage (novel feeder approach, predator response, immobility time, and time to capture), the ICC ranged from 0.002 to 0.45, suggesting that the cage-to-cage variance was never the majority source of variability. Further, variation in flock-level disease prevalence among cages (‘high’ versus ‘low’; see electronic supplementary material, table S4) did not qualitatively or quantitatively influence the patterns for latency to feed or respond to predators (see electronic supplementary material). For the models with repeated observations within birds (disease severity and pathogen load), the ICC was 0.14 for disease severity and 0.3 for pathogen load, indicating that bird-to-bird variance was not the primary source of variation for these observations.

## 4. Discussion

Here we experimentally demonstrate that infected individuals benefit from group-living by increasing behavioural responsiveness to both foraging opportunities and potential predation threats, relative to single-housed infected individuals. While healthy and diseased birds both showed detectable behavioural benefits of group-living in most assays, diseased birds housed alone consistently showed the lowest and slowest rates of feeding relative to both healthy birds and diseased birds housed in groups. Similarly, when evading capture from a human or responding to a mock aerial predator, single-housed diseased birds showed slower responses to both predation threats relative to all other treatment groups. Notably, these differences in behaviour were not due to effects of social housing versus isolation on disease severity or pathogen load, which were identical for MG-inoculated birds across social treatments. Together, our results indicate that group-living particularly benefits infected house finches by improving behavioural responsiveness to foraging opportunities and predator threats, both of which likely have important consequences for individual survival during infection.

In our foraging assays, group-housed birds were significantly more likely than single-housed diseased birds to approach and eat from their normal feeder after a disturbance, as well as to discover and eat from a novel feeder type. This behavioural difference may have important consequences for the overall survival of house finches in the wild, which rely heavily on bird feeders to meet their energetic needs [39], particularly during winter [40]. Our findings indicate that isolated diseased birds, which occur commonly in the wild [41], are less able to respond quickly to disturbance at a feeder that they regularly use. For group-living house finches, flockmates may allow them to respond more effectively to regular disturbances (refilling of feeders, which can occur multiple times per week [42]) as well as novel or atypical situations, such as implementation of an unfamiliar feeder type or establishment of a feeder in an area for the first time. In a study of another gregarious songbird, house sparrows (*Passer domesticus*), it was found that birds in larger groups were able to innovate and solve problems more quickly in response to a novel environment than those in smaller groups [43]. In our novel feeding assay, healthy birds were significantly more likely to eat from the novel feeder before their diseased flockmates, which is consistent with the hypothesis that the benefits of group-living were largely driven by healthy birds in a given flock. Overall, our assays suggest that group-living helps individual house finches to overcome novel foraging situations, but this benefit may be particularly important for diseased individuals.

Our anti-predator assays also indicated that the benefits of group-living, here in terms of predator avoidance, are particularly important for diseased birds. Single-housed diseased birds were significantly slower than all other treatment combinations in their responses to two different perceived threats at two different points during infection—capture by a human during early infection and an aerial predator flyover during peak infection. While these findings align with previous work showing that diseased house finches display reduced anti-predator responses [13], our results demonstrate that social context plays a key role in modifying effects of disease on an individual’s response to predators. In fact, diseased birds housed in groups largely had response times that aligned with their healthy flockmates, suggesting that group-living can largely ameliorate the effects of disease on anti-predator behaviours. Because predation accounts for most MG-related mortality [21], augmented anti-predator responses by infected birds living in groups could have important impacts on the ability of birds to survive infection. MG infection typically leads to swelling of the conjunctiva, which can sometimes progress to reduced visual acuity or even temporary blindness [44]. Notably, even though there were no differences in disease severity between MG-inoculated birds in each social group treatment, group-housed diseased birds responded twice as fast to an aerial predator than single-housed diseased house finches. The equivalent pathogen loads harboured by infected birds, regardless of group-living treatment, further support the possibility that infected house finches are showing behavioural tolerance by behaviourally offsetting the per-pathogen fitness costs of infection.

The potential mechanisms underlying the higher behavioural tolerance of infected birds housed in groups cannot be directly elucidated by our study design and may include multiple, non-mutually exclusive mechanisms. One possibility is that diseased birds in a flock receive key cues from their groupmates that are not available to isolated, diseased finches. The results of our feeding order analysis, whereby healthy birds were significantly more likely to use the novel feeder prior to a diseased flockmate, are consistent with the possibility that diseased birds use social cues from their healthy groupmates to detect novel food sources. However, other possibilities could generate similar patterns in feeding order without the use of social cues. Thus, future studies should explicitly examine whether diseased birds use social cues from their healthy groupmates to respond more quickly to behavioural opportunities and threats, as well as what type of cues are primarily used. Another possible mechanism for the observed behavioural differences across social treatment and disease status is that group-housed birds might be masking the sickness behaviours commonly associated with MG infection. Consistent with this possibility, single-housed diseased birds spent significantly more time immobile after the predator flyover relative to group-housed diseased birds. These findings align with the suppression of sickness behaviour in social settings by another highly gregarious songbird species, zebra finches (*Taeniopygia guttata*), when exposed to a simulated infection [23]. Though we only quantified a proxy for sickness behaviours during our aerial predator assay, both foraging behaviours and predator responses can be impacted by the presence or absence of sickness behaviours. By suppressing sickness behaviours such as immobility during infection, house finches in flocks may be able to increase anti-predator responses and foraging opportunities, compared to their more isolated conspecifics.

One caveat of our study design is that, due to housing constraints, single-housed healthy birds maintained auditory contact (though no visual contact) with three other single-housed birds during the study. Thus, some of the observed behavioural differences between single-housed diseased versus healthy birds could have been driven, in part, by differences in access to auditory cues during the foraging assays, which were conducted in the home cage. However, results of the novel feeder assay suggest that auditory cues were not important drivers of behavioural responses to foraging opportunities. Had auditory cues driven behavioural responses to novel feeders, then single-housed healthy birds should have fed from the novel feeder at rates more similar to that of the group-housed healthy birds (of which 9/16 fed from the novel feeder during the assay). Instead, only 1/8 single-housed healthy birds fed at the novel feeder, despite the fact that three additional single-housed healthy birds had auditory contact with that single successful individual during the behavioural assay. Further, the housing differences in the home cage could not have influenced responses to the aerial predator assay, for which all cages were assayed in a separate isolated room. Nonetheless, our aerial predator assay showed similar results to those of the foraging assays, suggesting that any detected differences in behavioural responses to foraging opportunities were not driven by access to auditory cues for single-housed healthy birds.

Overall, our results suggest that group-living may act as a key form of behavioural tolerance for diseased house finches in the wild. These results might help explain previously demonstrated preferences for associating with a flock during experimental MG infection in house finches [24]. However, while our assays found that birds housed in flocks of five were significantly more likely to eat from a feeder, in the wild, infected house finches may not be able to consistently keep up with flocks of healthy conspecifics. In fact, relative to healthy birds, house finches with severe disease are observed more often in smaller flocks while at feeding stations [41,45]. Because diseased house finches are not actively avoided by healthy conspecifics, and in the case of diseased males, are preferred over healthy birds as feeding partners [46], the smaller observed flock sizes of diseased birds in the wild are unlikely to result from avoidance by healthy conspecifics and instead likely represent an inability of diseased birds to adequately move across the landscape [47]. Although severely diseased wild birds may not be able to successfully associate with flocks of the size used in this study, even having one other house finch close by while feeding may be beneficial from a foraging behaviour perspective. In a study of multiple species of common North American bird-feeder birds, Berberi and colleagues [48] found that having even one conspecific with them at a feeder can help individuals combat lost foraging opportunities due to interspecific competition. However, our study was limited to observing foraging and anti-predator behaviours in the presence or absence of any group members, with birds being either single-housed or group-housed with a stable flock of four conspecifics. Considering the large body of evidence that differing group sizes impact behavioural phenotypes (e.g. [22,43,49]) and work that has shown that infected house finches in the wild are seen more often with smaller flocks [41,45], future work should explore optimal flock sizes, as well as the benefits and costs associated with smaller or larger flocks, for diseased individuals. Further, future studies should explore whether the disease status of flockmates influences the benefits and costs of sociality for a given individual, as has been shown in three-spined sticklebacks [50]. Although we did not have sufficient numbers of flocks to robustly test this question, we used underlying differences in transmission speed to probe whether the four flocks that had relatively high disease prevalence (4–5 diseased birds per flock) at the time of behavioural assays differed in behavioural responses to our assays when compared to flocks with lower disease prevalence (2–3 diseased birds per flock). Interestingly, behavioural responses at the flock level did not appear to vary with flock disease prevalence. However, future work should examine if both the number of individuals in a social group and their infection status contribute to differences in behavioural tolerance.

Our results show that group-living extends both foraging and anti-predator benefits to individual members, and these benefits are particularly strong for infected individuals. These results align with recent work showing that personality traits of individuals can mediate the benefits of social cues, with slow-exploring red knots benefiting more strongly from group foraging than fast-exploring individuals [51] Together, this underscores the importance of understanding how traits of individuals (disease status, personality, etc.) drive the relative behavioural benefits of group-living. Further, although not quantified here, the foraging and anti-predator benefits of group-living are likely synergistic to some extent. Increased foraging due to dilution of time spent on predator vigilance has been documented in multiple systems [9,22], so it is likely that fitness benefits provided by groups through increased predator defence and increased foraging success are not mutually exclusive. Thus, maintaining social groups during infection may provide multiple and overlapping benefits to individuals. While our study was conducted on a specific songbird species, the benefits detected here for infected house finches may be similar for other gregarious taxa, such as herbivorous [9] and cooperatively hunting mammals [10]. In other social systems, however, group-living during infection may come with added costs rather than benefits [52]. Such costs of sociality for some hosts during infection may help explain why many social animals actively or passively reduce their degree of social interaction when infected or expressing sickness behaviours (e.g. [53–56]).

In summary, our results show that infected house finches benefit directly from group-living, often to a stronger extent than healthy individuals. While such benefits may be key for individuals to survive infection in the wild, group-living of infected hosts will also directly augment transmission within the group (as seen for the initially healthy 32 flockmates, which showed 50% disease prevalence by the end of the study), representing a key cost for healthy group members. Thus, group-living during infection as a form of behavioural tolerance may, in turn, favour the evolution of counterstrategies in healthy individuals, such as the ability to detect and avoid (or show aggression towards) infected group members [57]. Overall, understanding the ways in which pathogen infection alters the key costs and benefits of group-living is critical for ultimately predicting both the ecological and evolutionary dynamics of pathogens in gregarious taxa, and the evolution of social behaviour in the face of rapidly changing natural enemies.

## Data Availability

Data and relevant code for this research work are stored in GitHub: https://github.com/housefinch42/GroupLiving25 and have been archived within the Zenodo repository [58]. Elecronic aupplementary material is available online [59].
